# Roles of systemic CD4^+^ T cells and local mast cells in a porcine model of eosinophilic esophagitis

**DOI:** 10.21203/rs.3.rs-9323196/v1

**Published:** 2026-04-28

**Authors:** Lizette M. Cortes, Tobias Käser, Joshua B. Wechsler, Anthony Blikslager, Evan S. Dellon

**Affiliations:** North Carolina State University; University of Veterinary Medicine; Lurie Children’s Hospital and Northwestern School of Medicine; North Carolina State University; University of North Carolina School of Medicine

**Keywords:** Porcine Animal Model, Eosinophilic Esophagitis (EoE), Mast Cells, CD4+ T Cells, Eosinophils, Esophageal Eosinophilia

## Abstract

Large-animal models are valuable for investigating the pathogenesis, natural history and therapeutic responses of eosinophilic esophagitis (EoE). We previously developed a porcine EoE model using hen egg white protein (HEWP) sensitization and oral challenge, but additional immunophenotyping is needed – particularly of esophageal mast cells (MC) and systemic CD4 T-cell responses. This study evaluated whether the model induces MC infiltration into the esophagus and whether systemic allergen-specific CD4 T cells correlate with esophageal eosinophilia. Pigs underwent three weekly intraperitoneal sensitizations with HEWP plus cholera toxin followed by one week of daily oral HEWP. Controls included: mock (n=3); sensitization only (n=3); and challenge only (n=3). For EoE induction, nine animals received both sensitization and challenge. MCs were identified by tryptase immunohistochemistry and peak MC counts per high power field (hpf) were manually quantified in the epithelium, lamina propria and muscularis. Systemic HEWP (ovalbumin, OVA)- specific CD4 T cells were quantified via flow cytometry and eosinophilic infiltration was determined by H&E staining. Correlations were analyzed using Spearman’s test. Sensitized and challenged pigs exhibited significantly elevated MC infiltration across all esophageal layers compared to controls. MC increases were mild in the epithelium (4.4 ±3.5 MCs/hpf vs 1.7 ±2.4; p=0.0064) but pronounced in the lamina propria (17.9 ±7.8 vs 9.1 ±7.2; p=0.0001) and muscularis (17.2 ±8.0 vs 12.1 ±11.8; p=0.0002). Moreover, systemic OVA-specific CD4 T-cell frequency positively correlated with esophageal eosinophil counts (R2=0.51; p=0.001). These findings support that our EoE model recapitulates the pathogenesis of human EoE as a type-2 inflammatory disease with multicellular infiltrate of eosinophils and MCs.

## Introduction

Eosinophilic esophagitis (EoE) is a chronic allergen-induced, type-2 immune-mediated disease of the esophagus characterized by symptoms of esophageal dysfunction and an eosinophilic predominant infiltrate in the esophagus [1]. In patients meeting clinical criteria for EoE, the diagnosis is confirmed by endoscopic esophageal biopsy showing a peak count of at least 15 eosinophils per high-power-field (eos/HPF) in the squamous epithelium, in the absence of other competing causes of eosinophilia [2].

While eosinophils are the hallmark of EoE for diagnosis and disease surveillance, mast cells and CD4 T cells play an important role in EoE pathogenesis [3]. Animal models of EoE have played a crucial role toward improving the understanding of mast cells and CD4 T cells [4]. Due to their relatively low cost, ease of maintenance, and high reproduction rate, mice are the predominant animal utilized to model inflammatory diseases, including EoE. However, murine models frequently lack key clinical signs or pathological changes representative of human disease, in particular for gastrointestinal diseases with potential pitfalls for EoE [4]. In contrast, pigs can be used as a relevant animal model for EoE given the anatomical and physiological similarities to humans and the ability to perform standard upper endoscopy creating a unique opportunity to assess treatment response [5].

We previously developed a porcine EoE model with hen egg white protein (HEWP) allergen sensitization and oral challenge [6–8]. This model showed esophageal eosinophilic infiltration and increased expression of eotaxin and genes related to allergic inflammation. However, the immunophenotype involving local mast cells and disease-correlation to the systemic CD4 T-cell response has not been characterized. Therefore, the purpose of this study was to determine whether a porcine EoE model results in esophageal mast cell infiltration and determine the degree of correlation between CD4 T cells and esophageal eosinophilia. We hypothesized porcine EoE is associated with increased mast cell counts throughout the esophagus, systemic CD4 T-cell proliferation and their correlation with esophageal eosinophilia.

## Results

### 3.1 Mast cell infiltration in the porcine EoE model

**Figure 1:**
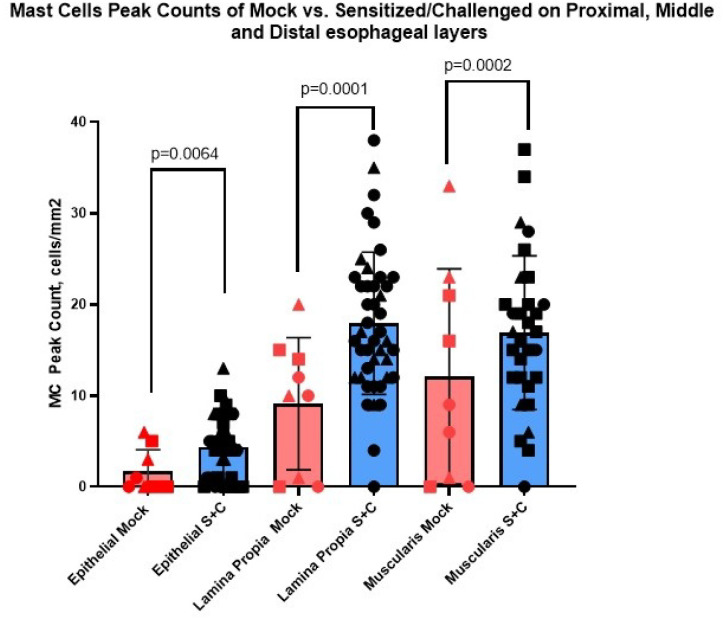
Esophageal tissue mast cells were significantly elevated in all esophageal compartments in the S+C pigs. Full thickness esophageal resection specimens were immunohistochemically stained for mast cells with anti-porcine tryptase. Peak mast cell count (HPF), for each compartment (esophageal epithelium, lamina propria, and muscularis) in the sensitized and challenged (S+C) porcine EoE model vs the mock pigs are here displayed.

We initially quantified mast cell infiltration into the esophagus. Compared to the three mock pigs, mast cell infiltration was significantly elevated in the nine S + C treated pigs in all three compartments (Fig. 1). While the increase of mast cells in the epithelium was significant, mean peak mast cell numbers quantified by MC/hpf (0.24mm2) stayed mostly below ten (4.4 ± 3.5 mast cells/hpf vs 1.7 ± 2.4; p = 0.0064). In contrast, mast cell infiltration in the lamina propria and muscularis were mostly above ten mast cells per square millimeter – lamina propria (17.9 ± 7.8 vs 9.1 ± 7.2; p = 0.0001) and muscularis (17.2 ± 8.0 vs 12.1 ± 11.8; p = 0.0002).

**Figure 2:**
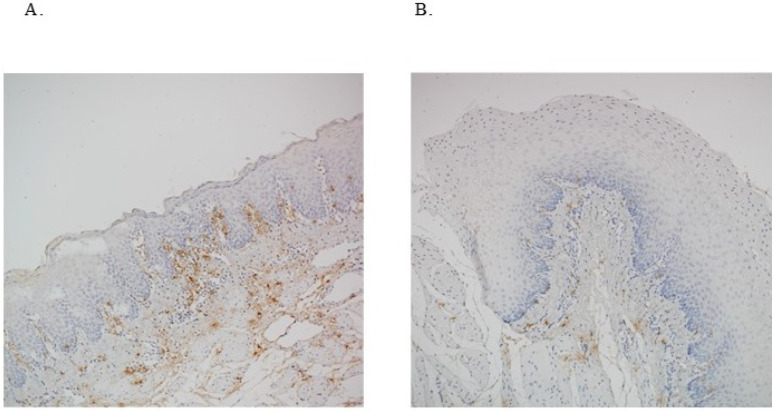
Representative example of tryptase staining in active EoE or control pigs. Full thickness esophageal resection specimens were immunohistochemically stained for mast cells with anti-porcine tryptase. In pigs with experimental EoE (S+C), expansion of the papillae into the epithelium was observed with mast cells commonly located right at the basal edge suggesting potential interaction (A). In control pigs the papillae were not expanded (B).

To provide a better understanding of the location of infiltrated mast cells, representative pictures full thickness esophageal resection specimens are shown in Fig. 2. In the S + C pigs, expansion of the papillae into the epithelium was observed with mast cells commonly located right at the basal edge, suggesting potential interaction (Fig. 2a). In the mock pigs, the papillae were not expanded (Fig. 2b). There also appeared to be more background staining in the epithelial and LP regions of the experimental group suggesting increased degranulation. This was not likely due to technical issues, since the muscle layer did not show any differences in background staining. Also, we observed greater basal zone hyperplasia in S + C pigs but not in mock pigs. In contrast to controls, eosinophils from S + C pigs seem also to be located right “below” the epithelium as if waiting to interactwith multiple cell types including epithelial cells, mast cells, sensory nerve endings, Th2, ILC2 and APCs among others.

### 3.2 Correlation of a systemic CD4 T-cell response with esophageal eosinophil and mast cell infiltration

**Figure 3:**
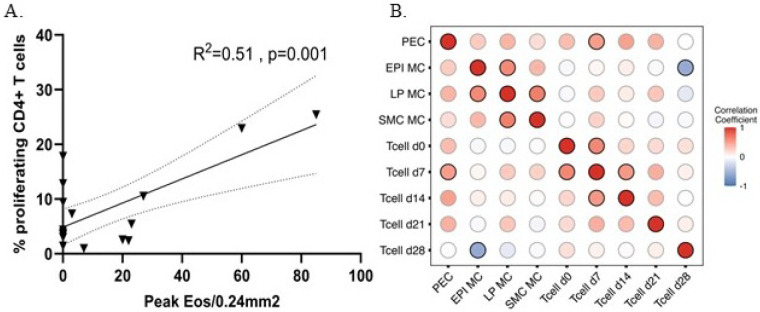
Correlation between peripheral blood CD4 T cells and peak tissue esophageal eosinophil counts in a porcine model of EoE. CD4 T-cell proliferation was analyzed at 0, 7, 14, and 21 dps; eosinophil and mast cell infiltration was studied at necropsy – 28 dps. A) Spearman correlation analysis between esophageal eosinophil infiltration (x-axis) and CD4 T-cell proliferation upon in vitro OVA restimulation at 7 dps (y-axis) is shown. B) Spearman correlation performed between all immune measures with color gradient used to denote correlation coefficient. Significant correlations with black border (p<0.05).

**Figure 4:**
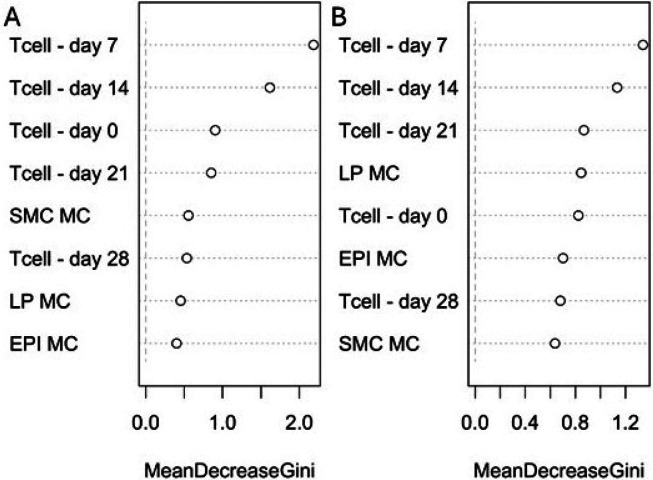
Random Forest demonstrates predictive value of T-cell proliferation in swine EoE. Random Forest was used to assess predictive contribution of T-cell proliferation and MC infiltrate for A) Sensitization + Challenge or B) Active EoE. Mean decrease Gini shown which demonstrates feature contribution to homogeneity of nodes/leaves within the decision trees

While local esophageal eosinophil infiltration is used to diagnose EoE, it is immunologically induced by a systemic CD4 T-cell response [3, 12–14]. To better understand this immunological connection in our model, we correlated the allergen-specific systemic CD4 T-cell response with esophageal eosinophil infiltration (Fig. 3). There was a moderate and significant positive correlation between peripheral blood CD4 T-cell proliferation at day 21 post-challenge and peak esophageal tissue eosinophil count across all animals (controls and S + C pigs, R^2^ = 0.51; p = 0.001, Fig. 3A). There was also a modest and significant correlation between mast cell infiltration in epithelium and systemic CD4 T-cell proliferation at day 28 post-challenge (R^2^ = 0.26; p = 0.044, Fig. 3B). However, there was no correlation between mast cell infiltration in lamina propria or muscularis with eosinophil infiltration or systemic CD4 T cells (Fig. 3B). To understand the extent to which T-cell proliferation and mast cell infiltrate best explained the group differences, we performed random forest using the T-cell proliferation percent and peak MC counts (Fig. 4). We identified CD4 T-cell proliferation at day 7 as the most predictive of both sensitization + challenge or active EoE.

## Discussion

EoE is a human esophageal disease that is rapidly increasing in incidence and prevalence, but much is still not known about EoE pathogenesis [15, 16]. Murine models of EoE exist and have been key for understanding EoE mechanisms. However, large animal models that can reproduce the immunopathogenesis of EoE are critically needed as they would allow endoscopic surveillance using standard human equipment, early testing of drugs and esophageal formulation delivery systems, and rapid translation to humans. The porcine esophagus is particularly well-suited for this work given its homology in structure and function to the human esophagus [17, 18].

In this study, we expand the immunophenotypic characterization of our previously established porcine EoE model [6–8] by evaluating esophageal mast cell infiltration and systemic CD4 T-cell responses, two features that play an important role in human EoE but that have not been fully examined in large animal models. Our results suggest that sensitization combined with oral challenge induces a robust, multilayer inflammatory response in pig esophageal tissue that mirrors characteristics of human disease. We focused our first assessment in this study on mast cells. In human EoE, mast cells are highly involved in EoE pathogenesis [19, 20]. These cells are increased compared to controls, are involved in fibrogenic pathways, smooth muscle dysregulation, and tissue remodeling, all of which lead to clinical symptoms of dysphagia and signs of esophageal stricturing [21–25]. Mast cell-related gene expression is also dysregulated in EoE, mast cells are associated with clinical features, and a subset of EoE patients have more mast cells than eosinophils [26–29]. For a porcine model of EoE to recapitulate the human disease, it is critical to demonstrate mast cell involved, which the present study shows. Notably, we found that this involvement is very early in disease pathogenesis - at just 28 days of antigen challenge. Since diagnosis of EoE in humans is usually delayed, it is yet to be demonstrated if these events also take place that early in human patients [30].

Moreover, we found that systemic allergen-specific CD4 T-cell activation correlates with esophageal eosinophil infiltration, supporting the concept that EoE represents a tissue-localized manifestation of a systemic Th2 immune response. Pathogenic effector T-helper type 2 (peTh2) cells are known to proliferate in patients with EoE. This is largely driven by antigens and support this model as directly relevant to human EoE [31]. Notably, we found CD4 T-cell proliferation correlated with intra-epithelial esophageal eosinophilia, and we hypothesize that peTh2 cells are a key source of cytokines like IL-4/5/13 all of which contribute to esophageal eosinophilia. IL-5 promotes bone marrow release of eosinophils and primes eosinophils for activation, while IL-4/13 drive release of eotaxin from the esophageal epithelial which recruit eosinophils [14]. Further studies to understand the extent to which peTh2 in swine EoE reflect human peTh2 are critical and will facilitate drug development studies [31].

Future studies in swine EoE would be valuable to explore the extent to which this HEWP-driven EoE model is ideal to further assess CD4 T cell-driven inflammation in the esophagus and how to impair this process. In addition, since IHC does not fully capture MC activation states, and peripheral CD4 T-cell proliferation may not fully reflect tissue-level activity, these limitations will be key targets for future studies. Other study limitations include the relatively small sample size and the short time frame of disease duration, which limited our ability to comment on longer-term complications (such as strictures) and assess treatment effects on these cell populations. We also acknowledge that multiple other cell types are involved in EoE pathogenesis which can be assessed in a high level of detail with techniques like single cell sequencing and special transcriptomics. All of these are planned future directions in our current research program.

In conclusion, we identified a key role for mast cells and CD4 T cells in experimental swine EoE. In addition to our previous findings of increased esophageal eosinophilia and endoscopic findings, our model continues to demonstrate important similarities with human EoE. In the future, this model system holds unique potential to continue to understand EoE pathogenesis and characterize other cell types and inflammatory pathways, study development of fibrostenosis, and assess therapeutics and outcomes.

## Materials and Methods

### 5.1 Swine model

The present study analyzes data and samples from a prior cohort of 18 pigs – 9 control pigs and 9 treated pigs. Control pigs received either mock treatment (MOCK, n = 3), sensitization only (SENS, n = 3), or oral challenge only (CHALL, n = 3). Experimental pigs received both, sensitization and oral challenge (S + C, n = 9). Treatment details have been published and are openly available [6]. In brief, sensitizations were performed three times by weekly intraperitoneal injections of either PBS (for controls) or 500 μg HEWP and 10 μg cholera toxin – at 0, 7, and 14 days post first sensitization (dps) [6–8]. From 21 to 28 dps, oral challenges took place by hand-feeding a daily dose of 10 g of HEWP mixed into commercial maple syrup and swine chow. Non-challenged swine received commercial maple syrup mixed into swine chow without HEWP. Starting at 0 dps, blood samples were collected weekly for PBMC and serum isolation. At the end of the study (28 dps), pigs underwent endoscopy to assess visible esophageal findings and to obtain esophageal mucosal biopsies. For this procedure, pigs were sedated by intramuscular injection of an anesthetic combination of Telazol (4 mg/kg), ketamine (5 mg/kg), and xylazine (2 mg/kg) (TKX). Right at the conclusion of these procedures, pigs were euthanized and necropsy was performed to collect full-thickness esophageal resection specimens, which are the focus of the present study. Prior to necropsy and euthanasia, deep anesthesia was confirmed, and euthanasia was performed by intravenous administration of sodium pentobarbital (100 mg/kg), followed by exsanguination as a secondary method to ensure death. Death was confirmed by the absence of heartbeat and respiration for at least 5 minutes, fixed dilated pupils, and absence of the corneal reflex. All experimental procedures were approved by the North Carolina State University Institutional Animal Care and Use Committee (IACUC; protocol ID #18–084-B; approval date: 25 May 2018) and were conducted in accordance with American Veterinary Medical Association (AVMA) guidelines and complied with the ARRIVE guidelines.

### 5.2 Mast cell immunohistochemical staining and quantification

Full thickness esophageal resection specimens were immunohistochemically stained for mast cells with anti-porcine tryptase (Biomatik, CAU26570, 1:250) at the Histology Phenotyping Laboratory on a Biocare IntelliPATH Autostainer (Biocare Medical, Pacheco, CA). Quantification of at least 5 high power fields (hpf) were assessed on a Nikon 80i (hpf = 0.24mm^2^) in each of the epithelial, lamina propria and muscularis compartments to assess peak mast cell counts for each tissue level [9, 10].

### 5.3 PBMC isolation and the OVA-specific CD4 T-cell response

The isolation of PBMCs was performed from whole blood by density centrifugation using Ficoll-Paque-Premium (GE Healthcare, Uppsala, Sweden) in SepMate tubes (Stemcell Technologies, Vancouver, Canada). After isolation, PBMCs were either used fresh for in vitro restimulation assays or frozen in 10% DMSO, 40% FBS, and 50% RPMI-1640 and stored in liquid nitrogen for further downstream analysis. For the analysis of the CD4 T-cell response, PBMCs were stained with CellTrace^™^ Violet proliferation dye (BV421, ThermoFisher). Then, stained PBMCs were cultured in 96-well plates at 2 × 10^5^ cells/well in the absence or presence of 50 μg/mL OVA. After 4 days, PBMCs were stained for flow cytometry using a directly conjugated anti-CD3-FITC antibody (clone PPT3, FITC, Southern Biotech), or an indirect staining strategy with an anti-CD4 primary antibody (clone 74-12-4, BEI Resources) and a secondary anti-mouse IgG2b antibody conjugated to BV480 (Jackson Immunoresearch). Dead cell exclusion was performed using the Invitrogen^™^ LIVE/Dead^™^ Near-IR Dead Cell Stain Kit according to manufacturer’s instructions. Data were acquired on a Beckman Coulter CytoFlex and analyzed by FlowJo v10.5.3.

### 5.4 Esophageal tissue eosinophil count determination

During necroscopy, the pig esophagus was removed en bloc, divided into thirds (proximal, mid, and distal), and tissue sections from each area were fixed in 4% formalin (Thermo Fisher Scientific, Waltham, MA) for 24 hours. The sections were embedded in paraffin and 5 μM sections were stained with hematoxylin and eosin (H&E). Microscopic images were taken on a BX41 light microscope (Olympus, JPN) equipped with a high-resolution 14MP MU1400B digital camera imaging system and the AmScope v4.8 image analysis software (AmScope, ToupTek Photonics, CN). Stained tissue samples were scored by a veterinary pathologist who was blinded to experimental groups. Esophageal eosinophil infiltration was determined by H + E staining. Based on the criteria to score human EoE [9, 10], eosinophil infiltration was quantified per histologic section of esophagus with a high-power field (eos/HPF; HPF size = 0.24 mm^2^), and the peak eosinophil count was recorded for each esophageal level (proximal, mid, distal) and in each esophageal wall layer (epithelium, lamina propria, and muscularis).

### 5.5 Statistical analysis

Statistical analysis was performed using GraphPad Prism 9.4.0 (GraphPad Software, San Diego, CA). Comparisons of mast cells per hpf were performed for each compartment between sensitized/challenged (S + C) and control groups. Multiparametric analysis was performed with Spearman correlation from peak eosinophils and CD4 T-cell proliferation at 21 dps. Random forest analysis was performed on R (4.5) using the *randomForest* module [11] with % CD4 T-cell proliferation at 0, 7, 14, 21, and 28 dps as well as peak mast cell counts from the epithelium, lamina propria and muscle layer as inputs. A p < 0.05 was considered significant.

## Data Availability

The datasets generated and/or analyzed during the current study are not publicly available, as we are planning further publications using these datasets; however, they are available from the corresponding author upon reasonable request.
